# Ultrasound-guided adductor canal block using levobupivacaine versus periarticular levobupivacaine infiltration after totalknee arthroplasty: a randomized clinical trial

**DOI:** 10.1590/1516-3180.2018.0269101218

**Published:** 2019-05-08

**Authors:** Faruk Cicekci, Ahmet Yildirim, Özkan Önal, Jale Bengi Celik, Inci Kara

**Affiliations:** I MD. Assistant Professor, Department of Anesthesiology, Selçuk Üniversitesi Tıp Fakültesi, Konya, Turkey.; II MD. Assistant Professor, Department of Orthopedics and Traumatology, Selçuk Üniversitesi Tıp Fakültesi, Konya, Turkey.; III MD. Associate Professor, Department of Anesthesiology, Selçuk Üniversitesi Tıp Fakültesi, Konya, Turkey.; IV MD. Professor, Department of Anesthesiology, Selçuk Üniversitesi Tıp Fakültesi, Konya, Turkey.; V MD. Associate Professor, Department of Anesthesiology, Selçuk Üniversitesi Tıp Fakültesi, Konya, Turkey.

**Keywords:** Levobupivacaine, Arthroplasty, replacement, knee, Ultrasonography, Rehabilitation, Analgesia

## Abstract

**BACKGROUND:**

Both postoperative pain control and range of motion are important in total knee arthroplasty (TKA). However, in the literature, there is little comparison of peripheral nerve blocks and periarticular infiltration techniques using levobupivacaine. The aim of our study was to measure pain with visual analogue scale (VAS) and knee range of motion (ROM) between in patients undergoing adductor canal block (ACB) for TKA using levobupivacaine compared to periarticular levobupivacaine infiltration (PAI-L).

**DESIGN AND SETTING:**

Prospective randomized clinical trial in a university hospital.

**METHODS:**

Patients aged 40-85 years who underwent unilateral TKA were included; 39 were treated withperiarticular infiltration using 40 ml (0.125 mg) of levobupivacaine (PAI-L group); and 40 were treated with ACB using 20 ml of 0.25% levobupivacaine (ACB-L group). Postoperative pain scores at rest and during active physical therapy were assessed using a VAS, along with knee ROM in flexion and extension. In addition, 100-foot walking time results, total morphine consumption and time of first analgesia requirement were recorded postoperatively.

**RESULTS:**

VAS scores at rest and during active physical therapy and the total amount of morphine consumed were lower in the ACB-L group than in the PAI-L group (P < 0.05). In contrast, knee ROM in flexion and extension and 100-foot walking times were greater in the PAI-L group than in the ACB-L group (P < 0.05).

**CONCLUSION:**

ACB-L was superior to PAI-L regarding pain treatment after TKA; however, PAI-L was superior to ACB-L regarding postoperative ROM and walking ability.

**CLINICAL TRIAL REGISTRY:**

ACTRN-12618000438257.

## INTRODUCTION

Total knee arthroplasty (TKA) is a common surgical procedure that can cause severe postoperative pain.^1^ Various methods for postoperative analgesia management are available, such as systemic opioids, epidural local anesthetic, peripheral nerve block and local anesthetic infiltration analgesia.^2^ Use of systemic opioids can cause adverse effects that may affect functional rehabilitation, such as nausea, vomiting, pruritus, sedation and respiratory depression.^3^^,^^4^ Hypotension,urinary retention, and pruritus are more common in patients with epidural analgesia.^5^ In addition, use of long-acting intrathecal opioids causes adverse effects such as bilateral motor block, tremor and hypotension.^6^ Systemic and intrathecal methods for postoperative analgesia are gradually being abandoned because of these negative effects.

Anesthesia management involving multimodal analgesic regimens, including regional anesthesia techniques such as femoral nerve block (FNB) and local infiltration analgesia (LIA), is commonly used for TKA.^3^^,^^6^ Although FNB is widely used in TKA, it can cause weakness of the quadriceps muscle and require use of a knee immobilizer, which may prevent early ambulation and can delay discharge.^7^^,^^8^

The saphenous nerve is the largest contributor to sensory perception around the knee, while the adductor canal contains the nerve to the vastus medialis, the medial femoral cutaneous nerve,the medial retinacular nerve, articular branches from the posterior division of the obturator nerve and occasionally the anterior branch of the obturator nerve.^9^^,^^10^ Although adductor canal block (ACB) can contribute towards motor blockade of the periarticular musculature, its effect on functional weakness of the quadriceps has been reported to be minimal, compared with FNB.^10^^,^^11^

One alternative analgesic technique for TKA is periarticular infiltration (PAI) using local anesthetic.^12^^,^^13^ Periarticular infiltration is commonly used because of its simplicity, but its efficacy for diminishing postoperative pain after TKA is a matter of controversy.^14^^,^^15^

## OBJECTIVE

The primary aim of this study was to compare postoperative pain scores from periarticular infiltration using levobupivacaine (PAI-L) and ultrasound-guided adductor canal block using levobupivacaine (ACB-L). Its secondary aims were to compare knee ROM, total morphine consumption and a 100-foot walking test data among patients undergoing elective unilateral TKA.

## METHODS

This randomized clinical study was reviewed and approved by the Necmettin Erbakan University, Medical Faculty Ethics Committee (reference no. 27.09.2017/146) and was registered in the Australian New Zealand Clinical Trial Registry: ACTRN-12618000438257. After obtaining the participants’ written informed consent, 94 patients aged 40-85 years whose American Society of Anesthesiologists (ASA) status was I-III and who had been scheduled to undergo unilateral TKA to treat degenerative joint disease were enrolled in the study.

The exclusion criteria were ASA status IV-V, age under 40years or over 85 years, presentation of chronic pain syndrome or neuropathic pain, morbid obesity, presence of coagulation disorder, inability to walk without help due to known knee disease, and presence of allergic reactions to the medications used in the study. Anotherexclusion criterion was initially set as consumption of more than 5 mg/day of oral morphine or equivalent opioids. However,this was subsequently changed (after study registration) because patients did need larger amounts of morphine, so this ceased to be a reasonable exclusion criterion. Therefore,even patients consuming larger amounts of morphine were analyzed in this study.

Participants who were eligible for inclusion in the study were identified from the records before the date of the orthopedic surgery. During the preoperative anesthesia examination, these patients were interviewed. They were invited to participate in the study and they filled out an informed consent form.

A computer-generated randomization sequence was prepared by our statisticians. Each study patient was assigned a study number. To avoid loss of concealment, the group to which each patient was allocated could only be accessed by the researchers after each patient had been registered for surgery. However, since the study groups included analgesia applied in different manners (ACB and PAI), the data thus collected could not be blinded. Two anesthetists performed ACB and PAI, while two other anesthetists collected the data.

### Anesthesia and surgical technique

The patients were admitted to the operating room without any premedication. Spinal anesthesia was administered by means of a 22-gauge spinal needle (Atraucan, Braun Germany), consisting of 15 mg (3 ml) of 0.5% spinal bupivacaine (Marcaine, Abbott Laboratories, Chicago, IL, USA) to each patient after both ACB and PAI. All operations were performed by the same surgical team using a similar technique.

### Intervention in the ACB-L group

Ultrasound-guided ACB was performed using a linear probe (10-18 MHz) (Esaote MyLab 30 US, Florence, Italy). The probe was placed midway between the inguinal ligament and the medial condyle of the knee under aseptic conditions, with the patient in the supine position, the knee slightly externally rotated and the leg outstretched (frog-leg position). An ultrasonographic image of the saphenous nerve was captured in the adductor canal, laterally to the femoral artery under the sartorius muscle. After negative aspiration, 20 ml of 0.25% levobupivacaine (Chirocaine, Abbott, Elverum, Norway) was administered into the nerve sheath using a 20-gauge, 100-mm, sloped, Teflon-coated unipolar needle (StimuplexUltra 360, B. Braun, Melsungen, Germany). The spreading of the local anesthetic spread in the adductor canal was viewed using ultrasonography.

### Intervention in the PAI-L group

A total of 40 ml (0.125 mg) of levobupivacaine was infiltrated into the posterior capsule, femur and tibia, medially and laterally to the joint capsule; and into the quadriceps tendon, vastus medialis obliquus, patellar tendon and dermal-epidermal junction.

### Postoperative protocol

Each patient received 50 mg of IV dexketoprofen (Arveles, Ufsa, Istanbul, Turkey) in the recovery room. Use of a patient-controlled analgesia (PCA) device for morphine delivery was started, and morphine consumption was recorded at 24 and 48 hours. Antibiotic prophylaxis was continued, consisting of 3×1 g cefazolin (Cefozin, Bilim, Istanbul, Turkey) for 24 hours. The patients were given dexketoprofen every 12 hours and a 1 g paracetamol tablet (Paranox, Sanofi, Istanbul, Turkey) every 8 hours until discharge. Twenty milligrams of IV metoclopramide (Metpamid, Recordati, Istanbul, Turkey) was given only to patients with nausea and vomiting. All patients were fitted with a knee immobilizer between 12 and 24 hours after the operation, until quadriceps muscle function was restored. Physical therapy was started 24 hours after surgery. Use of a continuous passive motion machine (CPM) (Kinetec, Smith & Nephew, Memphis, TN, USA) in a room in the orthopedic department was started with an initial setting of 45 degrees. CPM was used to the patients on the second postoperative day, for two-hour periods. The patients were also encouraged to starting on active motion of the knee.

### Clinical evaluation

Sociodemographic and clinical data such as age, sex, weight, height, body mass index (BMI), ASA status, side operated, length of operation and duration of tourniquet use were recorded. Painlevels at rest and during active physical therapy were recorded during the preoperative period, 30 minutes after the operation and 2, 6, 12, 24, 36 and 48 hours after the operation, according to scores on a visual analogue scale (VAS). Postoperative maximal ranges of flexion and extension on the 1^st^, 2^nd^ and 7^th^ days, and in the 2^nd^ and 6^th^ weeks, were also recorded. In addition, total morphine consumption and a 100-foot walking test data during the preoperative period, at 24 and 48 hours after the operation and at the time of the first requirement for analgesic were recorded.

The sample size calculation was based on a pilot study that we conducted on sixteen patients (whose data were not included in the present study). In this prior study, the mean difference and standard deviation (SD) of the VAS scores 24 hours after the operation between the ACB and PAI groups were 0.40 and 0.19, respectively. From this, it was determined that 39 subjects would be required to reach an α value of 0.05 and a power of 85%. Moreover, based on data from a retrospective study by Perlas et al.,^16^ the primary outcome SD was assumed to be approximately 3.0. It was estimated that the attrition rate due to canceled surgery or reasons of late patient ineligibility could be up to 20% and, therefore, to account for this, the final sample size selected was n = 94 (47 per group).

The statistical analyses in this study were performed using the Statistical Package for the Social Sciences (SPSS) 20.0 software. Continuous variables were presented in the form of mean ± standard deviation or error. The Kolmogorov-Smirnov normality test was used to assess continuous variables. Group comparisons on the variables that showed normal distribution were performed using one-way analysis of variance. Mann-Whitney U variance analysis was used for discrete numerical variables that did not show normal distribution. Relationships between the categorical variables were determined by preparing crosstabs and using the chi-square (χ^2^) test. P < 0.05 was accepted as statistically significant.

## RESULTS

**Figure 1.**
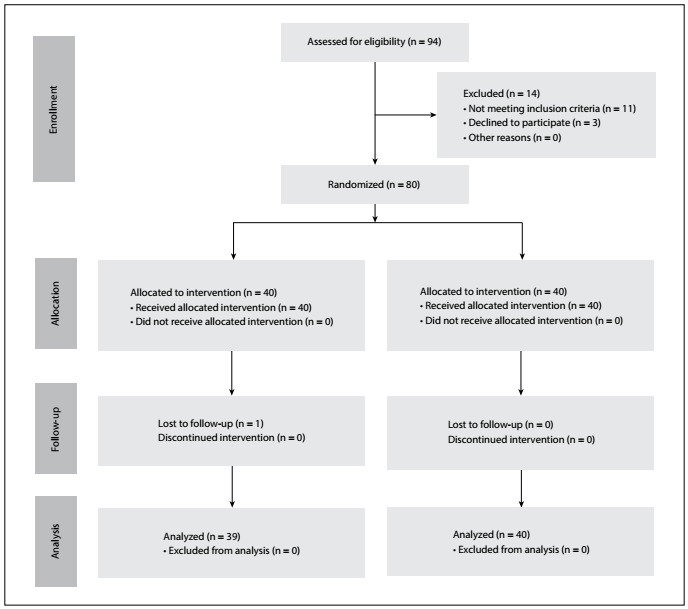
CONSORT flowchart diagram. ACB-L, adductor canal block using levobupivacaine; PAI-L, periarticular infiltration using levobupivacaine.

A total of 94 patients underwent elective unilateral TKA procedures between March 2017 and September 2017. A total of 14 patients were excluded for the following reasons: age over 85 years (n = 3); delay in admission to subacute rehabilitation (n=3); admission to the intensive care unit because of respiratory failure (n = 1); bilateral TKA was performed (4); and not wishing to participate (n = 3). Thus 80 patients were enrolled. One patient in the PAI group was further lost to follow-up because of non-attendance at follow-ups, and was therefore not included in the study analysis. Detailed information on enrollment of patients into the study is depicted in the CONSORT flow diagram in Figure 1**.** The patients’ demographic profiles and clinical characteristics were similar (P > 0.05) (Table 1).

**Table 1.** Patients’ characteristics

	ACB-L group n = 39	PAI-L group n = 40	P
Age (years)	69.1 ± 7.3	68.5 ± 7.5	0.697
Gender (F/M)	28/12	30/10	0.710
Weight (kg)	90.5 ± 10.5	88.4 ± 12.4	0.418
Height (cm)	165.9 ± 6.5	165.7 ± 6.4	0.904
BMI (kg/m^2^)	32.5 ± 1.2	32.0 ± 0.4	0.917
ASA (I/II/III)	3/31/6	2/29/9	0.586
Side of surgery (R/L)	22/18	23/17	0.823
Duration of surgery (minutes)	82.3 ± 16.9	84.9 ± 14.8	0.489
Duration of tourniquet use (minutes)	89.0 ± 16.6	90.8 ± 19.6	0.532

Values are presented as mean ± standard deviation or number of patients. ACB-L= adductor canal block using levobupivacaine; PAI-L = periarticular infiltration using levobupivacaine; F/M = female/male; BMI = body mass index; ASA (I/II/III) = American Society of Anesthesiologists status grade I/II/III; R/L = right/left.

**Figure 2.**
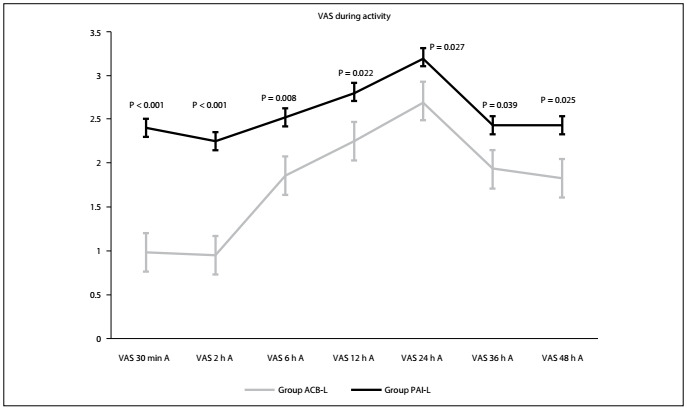
Comparison of postoperative visual analogue scale (VAS) scores during activity (A) between the two groups. There were statistical differences in VAS scores at all time points.

Over the first 48 postoperative hours, the VAS data at rest (mean ± standard error) for the ACB-L and PAI-L groups respectively were as follows: at 30 min, VAS 0.30 ± 0.08 and 1.38 ± 0.10 (P<0.001); at 2^nd^ hour, VAS 0.58 ± 0.11 and 1.35 ± 0.10 (P < 0.001); at 6^th^ hour, VAS 1.08 ± 0.11 and 1.92 ± 0.09 (P < 0.001); at 12^th^ hour, VAS 1.80 ± 0.14 and 2.38 ± 0.15 (P = 0.012); at 24^th^ hour, VAS 2.30 ± 0.16 and 2.80 ± 0.12 (P = 0.028); at 36^th^ hour, VAS 1.80 ± 0.10 and 2.35 ± 0.12 (P = 0.002); and at 48^th^ hour, VAS 2.00 ± 0.17 and 2.55 ± 0.12 (P = 0.016) (Figure 2).

**Figure 3.**
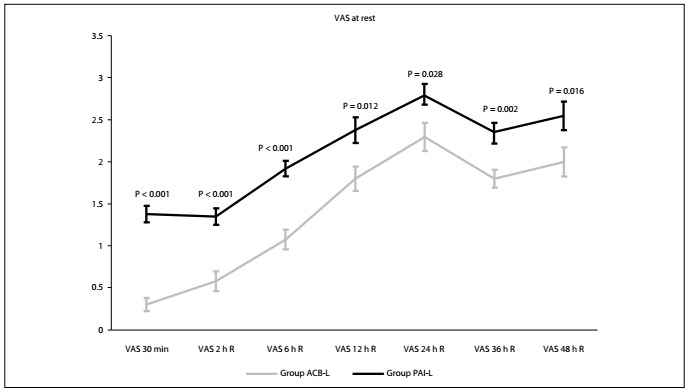
Comparison of postoperative visual analogue scale (VAS) scores at rest (R) between the two groups. There were statistical differences in VAS scores at all time points.

Over the first 48 postoperative hours, the VAS data with activity (mean ± standard error) for the ACB-L and PAI-L groups respectively were as follows: at 30 min, VAS 0.98 ± 0.09 and 2.40 ± 0.13 (P< 0.001); at 2^nd^ hour, VAS 0.95 ± 0.11 and 2.25 ± 0.13 (P<0.001); at 6^th^ hour, VAS 1.85 ± 0.13 and 2.52 ± 0.17 (P = 0.008); at 12^th^ hour, VAS 2.25 ± 0.15 and 2.80 ± 0.15 (P = 0.022); at 24^th^ hour, VAS 2.70 ± 0.15 and 3.20 ± 0.14 (P = 0.027); at 36^th^ hour, VAS 1.93 ± 0.19 and 2.43 ± 0.12 (P = 0.039); and at 48^th^ hour, VAS 1.83 ± 0.17 and 2.43 ± 0.12 (P = 0.025) (Figure 3).

With the exceptions of the preoperative scores and the postoperative 2^nd^ and 6^th^ week scores, the ACB-L group had less range of flexion and extension than the PAI-L group on the 1^st^, 2^nd^ and 7^th^ days after surgery. There were significant differences in range of flexion and extension between the groups (P < 0.05) (Table 2).

**Table 2.** Range of motion (ROM) in flexion and extension and time taken to perform 100-foot walking test before the operation and at different times after the operation, compared between the two groups

	ACB-L group n = 39	PAI-L group n = 40	P
Range of motion in flexion (degrees)			
Preoperative	109.2 ± 6.9	108.5 ± 6.7	0.625
Postoperative 1^st^ day	51.1 ± 6.4	69.3 ± 6.7	< 0.001
2^nd^ day	64.3 ± 6.2	86.3 ± 10.9	< 0.001
1^st^ week	96.2 ± 7.7	104.7 ± 11.9	< 0.001
2^nd^ week	121.7 ± 6.4	124.2 ± 5.7	0.071
6^th^ week	125.6 ± 6.8	125.6 ± 4.2	0.908
Range of motion in extension (degrees)			
Preoperative	4.3 ± 3.2	4.5 ± 3.3	0.866
Postoperative 1^st^ day	11.0 ± 3.6	8.3 ± 3.0	0.001
2^nd^ day	9.2 ± 2.6	4.8 ± 2.1	< 0.001
1^st^ week	4.2 ± 2.4	1.2 ± 2.1	< 0.001
2^nd^ week	0.3 ± 1.3	0.2 ± 1.1	0.649
6^th^ week	0.2 ± 1.1	0.2 ± 1.1	1.000
Time taken to perform 100-foot walking test (seconds)			
Preoperative	82.2 ± 12.3	79.1 ± 13.3	0.392
Postoperative 24^th^ hour	218.9 ± 33.9	192.2 ± 24.6	< 0.001
Postoperative 48^th^ hour	139.8 ± 19.5	112.0 ± 16.4	< 0.001

Values are presented as mean ± standard error. ACB-L = adductor canal block using levobupivacaine; PAI-L = periarticular infiltration using levobupivacaine.

Differently to the preoperative measurements, the time taken to perform the 100-foot walking test was significantly longer in the ACB-L group than in the PAI-L group at 24 and 48 hours postoperatively (218.9 ± 33.9 versus 192.2 ± 24.6 sec and 139.8 ± 19.5 versus 112.0 ± 16.4 seconds, respectively; P < 0.001) (Table 2).

The total morphine consumption was significantly lower in the ACB-L group than in the PAI-L group at 48 hours postoperatively (21.9 ± 8.9 versus 33.0 ± 9.5 mg; P < 0.001) (Table 3). The times of first requirement for analgesia in the ACB-L and PAI-L groups were 405.3 ± 41.0 and 316.7 ± 36.3 minutes, respectively. The difference between the groups was significant (P < 0.001) (Table 3).

**Table 3.** Time that elapsed until first requirement for analgesia and the total amount of morphine consumed over the ﬁrst 48 hours after surgery

	ACB-L group n = 39	PAI-L group n = 40	P
Time that elapsed until first requirement for analgesia (minutes)	405.3 ± 4.1	316.7 ± 36.3	< 0.001
Total amount of morphine consumed via PCA (mg)	21.9 ± 8.9	33.0 ± 9.5	< 0.001

Values are presented as means ± standard deviation. ACB-L = adductor canal block using levobupivacaine; PAI-L = periarticular infiltration using levobupivacaine; PCA = patient-controlled analgesia.

## DISCUSSION

In the present study, the ACB-L group had better postoperative analgesia both at rest and during active mobilization, compared with the PAI-L group over the first 48 hours after elective unilateral TKA. In addition, less morphine consumption was seen in the ACB-L group. However, during the first week, we found that the PAI-L group presented better flexion and extension knee movements. Moreover, the PAI-L group achieved better results in the walking test than did the ACB-L group. A number of studies have compared use of PAI and nerve block by means of local anesthetic agents for pain control in TKA,^2^^,^^6^^,^^17^ but our study was the first to use levobupivacaine in comparing ACB and PAI.

The adductor canal contains several nerve branches that supply sensory innervations to the knee. These nerve branches consistently include the saphenous nerve (which innervates the infrapatellar skin and the anterior knee capsule) and a distal branch of the motor nerve to the vastus medialis (which provides sensory innervation to the superomedial aspect of the knee and the knee capsule).^16^ Ultrasound-guided ACB is used as a theoretical alternative to FNB because the latter has adverse effects such as quadriceps weakness, lack of early ambulation and limitation of physical therapy. With ACB, a rather pure sensory block is obtained instead of the motor block on the knee, while equivalent pain control is achieved.^9^^,^^18^

Periarticular local infiltration of anesthetic is one of the most important procedures in multimodal pain control protocols.^19^ This analgesic technique has been specially developed to provide early mobilization and discharge, to avoid sedation and to facilitate rapid physiological recovery after lower-limb arthroplasty. Contrary to femoral nerve block, periarticular infiltration does not inhibit quadriceps function and, at the same time, it reaches the posterior capsule of the knee joint.

Chaumeran et al. conducted a study on FNB and PAI using bupivacaine. They found that the VAS scores and ROM values at rest and in movement were similar, but that PAI gave better results than FNB over the walking distance.^20^

In recent studies on liposomal bupivacaine, the VAS scores, total morphine consumption and ROM values were found to be similar in the PAI and FNB groups until the 48^th^ postoperative hour.^17^^,^^21^^,^^22^ Yu et al. showed that FNB using liposomal bupivacaine provided better analgesia but less walking distance than did PAI.^23^

In a variety of studies that compared use of ropivacaine for ACB and PAI, the VAS scores at rest and during activity, the morphine consumption and the walking distance results were similar in the two groups until 48 hours postoperatively.^16^^,^^24^ Incontrast to the studies of Perlas et al.^16^ and Sawhney et al.^24^, our study found that the total morphine consumption was significantly lower than the ACB-L group at 48 hours postoperatively. In a study by Li et al. that compared three groups (ACB, FBN and PAI), these authors reported that the VAS values were the same at rest and during movement between the three groups, and that ACB and PAI were also the same in terms of muscle strength.^25^

Levobupivacaine is the S (-) enantiomer of bupivacaine, with less cardiac toxicity and motor block than bupivacaine, but longer duration of action.^26^ Kovalak et al. reported that use of continuous femoral nerve block (CFNB) gave rise to superior VAS scores at rest and during activity, better passive and active ROM, lower total opioid consumption and better two-minute walking test results than did use of PAI.^27^ However,in their study, levobupivacaine infiltration was administered to the knee joint capsule of all patients in both groups. In a study by Wall et al.,^28^ it was reported that the effects of levobupivacaine for FNB and PAI on postoperative VAS values were similar. The authors^28^ showed that ACB gave rise to better VAS scores at rest and during activity than did FNB, over the first 48 postoperative hours.

Preoperative range of motion is the biggest indicator of postoperative range of motion. Many factors determine the range of motion after surgery. Rehabilitation programs after total knee arthroplasty should not be halted until at least 90° of knee flexion has been achieved, so that patients can resume normal social life.^29^ Ritter et al. found that age, preoperative range of motion, intraoperative range of motion and posterior capsule relaxation during surgery were important. Theyexplained that after the first year, there was no further effect on the degree of flexion from the passage of time, and that the range of motion acquired in the first six months was important.^30^ Consequently,after knee prosthetic surgery, the preoperative range of motion, the degree of relaxation of the posterior capsule during surgery, the patient’s age and the etiology of osteoarthritis take on importance.

The ability of a patient to perform functional activities, such as walking, rising from a chair and climbing stairs, depends on sufficient postoperative knee ROM. Isometric quadriceps exercises are started on the first postoperative day in our service. Knee ROM exercises for the first 3 days 0-30° flexion, at least 90° knee flexion between days.^31^ A meta-analysis on the range of knee flexion that compared use of periarticular local infiltration with FNB did not find any significant difference between these two groups.^32^ However, another meta-analysis suggested that post-TKA patients who underwent ACB showed better outcomes regarding ROM than did those who underwent FNB, throughout the first 72 h (i.e. post-anesthesia and after 24, 48 and 72 hours).^33^ This latter meta-analysis showed that PAI provided better flexion and extension ROM values postoperatively on the 1^st^ and 2^nd^ days and after the 1^st^ week than did ACB^34^.

According to the common milestones used in relation to TKA treatments, patients who can walk 100 feet (= 30.48 meters) with an assistive device, go to the toilet, make transfers, perform basic daily activities and do home exercise programs independently are in a condition in which they can be discharged home. In the present study, the results from 100-feet walking tests over the first 48 postoperative hours were better with use of PAI than with use of ACB. A placebo-controlled randomized trial on ACB suggested that administration of ropivacaine into the adductor canal provided effective analgesia, such that it significantly reduced pain and improved postoperative mobility, compared with placebo.^24^

In our study, we found that the VAS scores were better and the total amount of morphine consumed was lower in the ACB-L group than in the PAI group. However, the ROM data and walking results were shown to be superior in the PAI-L group. These results can be explained by the blocking of the saphenous nerve in the adductor canal through use of levobupivacaine over the first 48 hours after the operation. However, a possible attenuating effect on quadriceps muscle strength from ACB also accounts for the knee ROM results and the walking results in the PAI-L group.^34^^,^^35^

The main limitation of the current study was the inability to blind both the participants and the physicians to comparisons between peripheral nerve blockade and periarticular injection. This lack of blindness may have introduced some risk of bias from both the patients and the physicians. The outcome assessments from the adjudicators and all the statistical analyses were conducted in a blinded manner. In addition, the impossibility of measuring quadriceps muscle power before and after the operation using special instruments was another limitation. If this had been possible, the evaluation between the ACB-L and the PAI-L groups could have been more objective.

## CONCLUSION

This randomized clinical trial found that after total knee arthroplasty, ultrasound-guided adductor canal block with levobupivacaine was associated with shorter time taken to perform 100-foot walking test (24 hours post-operatively) and lower post-operative consumption of morphine when compared to periarticular infiltration with levobupivacaine. However, no difference between these interventions was found for range of knee motion after six weeks, pain at rest after 48 hours and pain during activities after 48 hours.
